# Dystonia as a Presenting Feature of Acute Ischemic Stroke: A Case Report and Literature Review

**DOI:** 10.7759/cureus.17272

**Published:** 2021-08-18

**Authors:** Yoram A Roman Casul, Meghan L Humbert, Amreen Farooqui, Aparna Wagle Shukla, Nandakumar Nagaraja

**Affiliations:** 1 Department of Neurology, University of Florida College of Medicine, Gainesville, USA

**Keywords:** acute ischemic stroke, stroke, acute dystonia, dystonia, focal dystonia

## Abstract

Hypokinetic and hyperkinetic movement disorders can occur post-stroke. Of these, dystonia is known to occur in the chronic stage of stroke. Rarely, acute dystonia can present as a symptom of acute ischemic stroke or develop during hospitalization for ischemic stroke. In this article, we present a case of acute focal dystonia as a presenting symptom of acute ischemic stroke, review the literature to summarize previous reports, and provide more insight into the pathophysiologic mechanisms related to this presentation.

## Introduction

Dystonia is an abnormal involuntary movement consisting of sustained muscle contraction that frequently appears as abnormal posturing or twisting and repetitive movements. Dystonia is the second most observed phenotype of post-stroke dyskinesias representing around 20% of cases [1]. The other two phenotypes are choreiform dyskinesias (athetosis, ballism, chorea) and non-chorea-dystonic dyskinesias (myoclonus, asterixis, tremor) [2]. Post-stroke dyskinesias can vary in onset after acute stroke and length of course depending on the type. Nearly 50% of hemidystonia is accounted for by stroke which is the cause of most hemidystonia onset after the age of 50 [2]. Post-stroke dystonias tend to have a delayed onset ranging between three months and three years with an average of 9.5 months [2], while some have reported onset as early as day one of stroke [2,3]. In contrast, hemichorea can develop within just a few days (4.3 days on average) following a stroke. Prognosis varies depending on the location which can be cortical or basal ganglia including subthalamic nuclei [2,3]. Thus, dystonia as a presentation of acute ischemic stroke (AIS) is uncommon. Here, we present a case of acute focal dystonia as a presenting symptom of AIS, review the literature to summarize previous reports, and provide further insight into the pathophysiologic mechanisms related to this presentation.

## Case presentation

**Figure 1:**
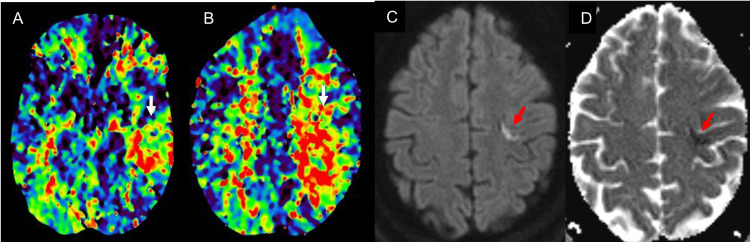
Increased time to peak (A, B) in the left posterior frontal, parietal, and temporal lobe; ischemic stroke on diffusion-weighted imaging (C); and apparent diffusion coefficient (D) in the left primary motor and supplementary motor cortex.

A 52-year-old woman with a past medical history of uncontrolled type II diabetes mellitus presented with right upper extremity weakness of one-hour duration. While communicating with the emergency department team, the patient had an episode of spontaneous painful posturing with rotation and flexion of the right arm and extension of the right leg that resolved in less than a minute without intervention. During evaluation by the stroke team, she had another episode of similar painful posturing. The National Institutes of Health Stroke Scale score was 2 for right upper extremity ataxia and dysarthria. She had mild weakness of the right wrist extensors without any right arm drift. Blood pressure on admission was 202/99 mmHg and blood glucose level was 358 mg/dL. CT head did not show acute hemorrhage or notable hypodensity. CT angiogram of the head and neck showed multifocal intracranial atherosclerosis particularly in the left middle cerebral artery and internal carotid artery. CT perfusion showed the increased time to peak involving the left posterior frontal lobe, parietal lobe, and posterior temporal lobe (Figure 1). Thrombolysis was done using intravenous alteplase. MRI brain showed small embolic strokes in the left primary motor and supplementary motor area (Figure 1) and a small lesion in the left parietal cortex. Stroke workup was remarkable for positive urine drug toxicology for cocaine, apical akinesia of left ventricle on transthoracic echocardiogram, hemoglobin A1c of 16.7%, total cholesterol of 378 mg/dL, high-density lipoprotein of 35 mg/dL, triglycerides of 422 mm/dL, and incalculable low-density lipoprotein. Stroke was determined to have two or more etiologies, namely, left middle cerebral artery stenosis, apical akinesia of left ventricle, and cocaine use. The patient was started on dual antiplatelet therapy and statin for secondary stroke prevention. She did not have further dystonia episodes during admission and was discharged home without any complications.

## Discussion

A systematic review was performed in compliance with the Preferred Reporting Items for Systematic Reviews and Meta-Analysis (PRISMA) statement [4]. A comprehensive search of the PubMed database was performed on March 14, 2021. The main keywords used in the search were “acute stroke, stroke, acute dystonic seizure, dystonia, acute dystonia.” All articles in PubMed from its inception were included in the search. The reference list of the articles that met the eligibility criteria was reviewed.

The inclusion criteria for the selection of articles were: (1) patient had a primary diagnosis of AIS, (2) patient presented with acute episodic dystonic movement or had dystonia during hospitalization for AIS, (3) there was a detailed description of the case along with supporting evidence related to the diagnosis. The exclusion criteria for the articles were: (1) presence of intracerebral hemorrhage on CT, (2) insufficient data supporting movement disorder as a presenting symptom in the acute phase, (3) dystonia occurring as a delayed manifestation more than one week after stroke onset.

Two authors (NN and YR) screened the titles and abstracts to identify articles relevant to the study. The selected articles were reviewed in detail (by MH) to extract relevant data using a data extraction form that included variables for demographics, clinical presentation, stroke location, stroke etiology, treatment received, and outcomes.

**Figure 2:**
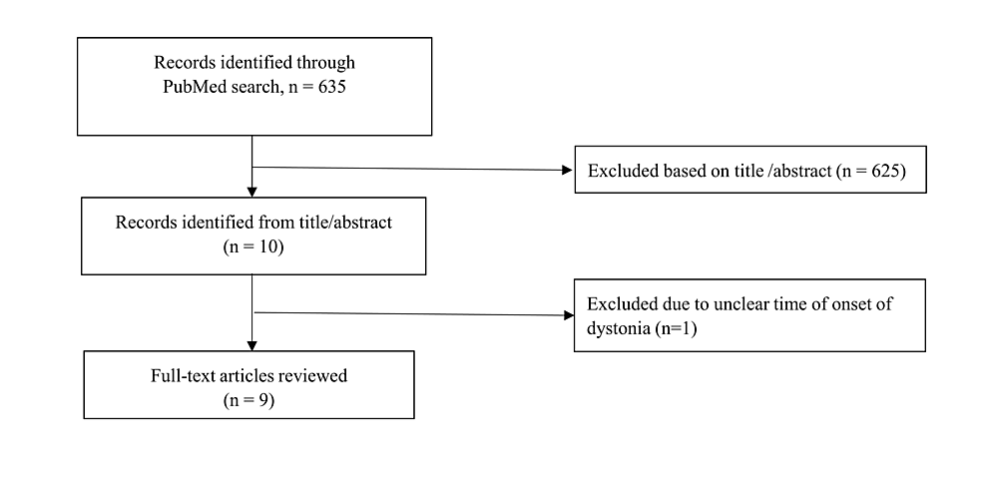
PRISMA flowchart. PRISMA: Preferred Reporting Items for Systematic Reviews and Meta-Analysis

A total of 635 articles were reviewed from PubMed with the study search terms. Among them, nine articles met the study criteria. There was a total of nine cases of acute dystonia presenting as AIS. Figure 2 shows the flow diagram of several articles identified and those included in the review. Cases were grouped in those presenting with (1) cortical lesions including those involving supplementary motor area (SMA) and premotor cortex, (2) subcortical lesions such as the thalamus and basal ganglia, and (3) brainstem (midbrain, pons, and medulla) and cerebellar lesions (Table 1).

**Table 1:** Clinical characteristics of acute dystonia in acute ischemic stroke.

Author, year	Age, gender	Clinical presentation	Stroke location	Stroke etiology	Treatment/Outcome
Dhakar et al., 2015 [5]	63, F	Presented with sudden-onset left upper extremity stiffness. Sustained muscle contractions causing flexion at the elbow, wrist, and fingers; left facial upper motor neuron weakness; and left lower extremity weakness without an increase in tone	Right premotor cortex and supplementary motor area	Intracranial atherosclerosis	Discharged to rehab, gradual improvement of dystonia
Nishimura et al., 2014 [6]	63, M	Presented with the stiffness of left limbs, dysarthria, and left central facial palsy. On day 2, the patient developed dystonic posture of the left upper extremity	Right supplementary motor area and left precentral gyrus	Left internal carotid artery occlusion at its origin	Dystonia improved spontaneously
Miletić et al, 2015 [7]	71, F	Presented with right hemiparesis and right supranuclear facial palsy that started 5 hours prior to admission. Hemidystonia in the right upper extremity occurred 18 hours after stroke onset	Left frontal cortical region	Cardioembolic (atrial fibrillation)	Haloperidol
Sabrie et al., 2015 [8]	25, M	Presented with left-sided hemiparesis, left-sided hypoesthesia with proprioceptive dysfunction, and left homonymous hemianopsia. Developed dystonia in his left arm on day 2 of stroke	Right thalamus and medial temporal lobe	Patent foramen ovale and antiphospholipid antibodies	Clonazepam (unsuccessful) followed by carbamazepine
Choi et al., 2015 [9]	66, M	Presented with a 3-week history of gait disturbance, difficulty writing, dysphagia, and dysarthria. On examination, the patient had multifocal dystonia in bilateral hands and feet as well as tongue contractions, bilateral ataxia in the upper and lower extremities, and wide-based gait	Right midbrain	Small-vessel disease	Clonazepam and trihexyphenidyl (no improvement)
Kim et al., 2009 [10]	56, M	Presented with headache, left hemiparesis, and dysarthria. On day 4, the patient developed dystonia in the form of left wrist sustaining choreiform movements, along with posturing of the arm	Right midbrain lesion with extension into the substantia nigra and subthalamic nucleus	Small-vessel disease	Unsuccessful trials of tetrabenazine, haloperidol, orphenadrine, and botulinum toxin. Eventually went for deep brain stimulation surgery
Tan et al., 2005 [11]	43, M	Presented with sudden-onset weakness and spasms of right hemibody, dysarthria, and mild right facial upper motor nerve palsy. Within hours of his initial presentation, he developed pain and spasms in his right upper and lower extremities	Left paramedian and ventral pons	Intracranial atherosclerosis	Clonazepam. Mild dystonia was observed at 1-year follow-up
Ogawa et al., 2018 [12]	86, F	Presented with weakness in the left upper and lower extremities, numbness of the right upper and lower extremities, and dysarthria. Several days following stroke, the patient demonstrated cervico-shoulder dystonia	Left lateral medullary infarction	Left vertebral artery occlusion	N/A
Zadro et al, 2008 [13]	48, F	Sudden-onset vertigo, vomiting, and ataxia. Horizontal, bidirectional nystagmus and ataxia of the left limbs. On day 2 of admission, the patient developed abnormal movement of the head with spasm of the cervical muscles	Left cerebellar infarction	Undetermined	Clonazepam and baclofen

Three patients with acute dystonia in AIS had cortical structures involved such as our patient. One patient presented with left upper extremity dystonia following right premotor and SMA stroke [5], while the other patient had acute dystonic posturing of his left upper extremity two days after initially presenting with left-sided stiffness and was found to have right SMA stroke [6]. Dystonia in these two patients resolved spontaneously without any acute intervention. Another patient presented with right hemiparesis and subsequently developed dystonia 18 hours after onset secondary to left frontal cortical ischemic stroke due to cardioembolic etiology [7]. The patient was started on haloperidol during admission for treatment of his dystonia. Two out of these three cases had facial, upper, and lower extremity paresis in addition to dystonia.

Subcortical structure such as the thalamus was involved in one patient who presented with left-sided weakness and acute dystonia on day two of stroke secondary to antiphospholipid syndrome and patent foramen ovale [8]. This patient was initially started on clonazepam without improvement and later transitioned to carbamazepine.

Although unexpected, there were cases of acute dystonia presenting as ischemic strokes involving brainstem structures. Of the five cases, two had strokes in the midbrain [9,10], and one each had strokes in the pons [11], medulla [12], and cerebellum [13]. Of particular interest was a 56-year-old male who was noted to have dystonic posturing on his left wrist four days after initial presentation with left-sided weakness and dysarthria. This patient also had involvement of substantial nigra and subthalamic nucleus supporting the idea of the dystonic phenomenon being secondary to disruption of motor pathways [10]. The patient’s dystonia was also very difficult to control requiring numerous medications without success and eventually needing deep brain stimulation as treatment [10]. One patient with cerebellar infarction who presented with vertigo, vomiting, nystagmus, and ataxia later developed cervical dystonia. There was a slight improvement in the patient’s condition with clonazepam and baclofen and a significant improvement with botox treatment a month later [13].

Post-stroke movement disorders can be either hypo or hyperkinetic, with post-stroke dyskinesias (involuntary hyperkinetic movements) the more common of the two to present in the first year following a stroke [1]. The prevalence of hyperkinetic movement disorders is uncommon, with some studies reporting a prevalence of about 1% (29/2,500) with an incidence of 0.08% per year [14]. A large portion of those with basal ganglia lesions did not result in movement disorders, which supports the rarity of post-stroke movement disorders and highlights that location is not predictive of whether patients will have movement disorders after stroke [14]. Dystonia and other types of abnormal involuntary movements such as chorea, tremors, and parkinsonism are commonly associated with both ischemic and hemorrhagic strokes [15].

Dystonia is a network disorder involving the motor cortex, where motor commands are generated, and the basal ganglia and cerebellum, where these are further refined to allow for smoother and more coordinated movements. There exists a balance between inhibitory and excitatory pathways. The “direct pathway” is the excitatory pathway resulting from the disinhibition of upper motor neurons via connections from the caudate and putamen to the globus pallidus internal segment and substantia nigra. The “indirect pathway” is responsible for modulating disinhibition of the direct pathway (a sort of check feature), and the net result is the activation of the indirect pathway that leads to inhibition of the upper motor neurons [16]. Although stroke-related dyskinesia pathogenesis is not yet completely understood, some suggestions include post-synaptic denervation hypersensitivity as well as impaired plasticity of axons and dendrites after an ischemic injury [1]. While rare, they can occur at any point in the motor circuitry. No one location in this pathway is a reliable predictor of if or which particular dyskinesia will arise, and hence, stroke-related movement disorders are not easy to predict based on stroke anatomical characteristics alone [1].

The most common site of isolated lesions causing dystonia is the putamen but the thalamus, pallidum, caudate, and midbrain have also been implicated [2]. The suggestion is to interrupt the connections between the basal ganglia, specifically to the sensorimotor part of the putamen, striatum, and pallidum, which increases thalamic drive projecting to frontal and motor cortical areas resulting in dystonia [2,17]. Other studies also support that hyperkinetic movements following stroke result from thalamic disinhibition, which, in turn, releases the motor cortex in the indirect pathway; a similar result is seen from overactivity of the direct pathway [1]. Efferent projections from the SMA to the putamen, caudate nucleus, and subthalamus likely contribute to spreading signals and dystonic symptoms [18]. In our study, only one patient who had dystonia in AIS had a lesion in the thalamus. Whereas the thalamus and basal ganglia are common sites implicated for delayed-onset dystonia, commonly seen post-stroke, they may be less likely involved in the acute presentation of dystonia in AIS.

It could be challenging to determine if a patient presents with isolated acute dystonia or a symptom of AIS. Based on our case and previous case reports, if a patient has any associated neurological symptoms such as weakness in the extremities, facial droop, or dysarthria, it should raise the possibility of AIS and should be considered in the differential diagnosis. Screening for thrombolytics should be considered if they are in the 0-4.5-hour treatment window. The presence of perfusion deficit on CT perfusion scan could suggest that it is an AIS rather than isolated dystonia, as in our case.

Our study had limitations. The review was limited to a literature search involving the PubMed database. Our study focused on AIS. Patients with intracerebral hemorrhages were excluded. We also focused on articles that described dystonia as a presenting symptom of stroke or that developed during hospitalization. Articles that reported dystonia as a chronic effect of stroke or those that did not describe the timing of dystonia in relation to stroke onset were excluded.

## Conclusions

Although not a common presentation, acute dystonia can be a presenting symptom of AIS. It occurs in stroke involving the frontal lobe and brainstem structures and is probably less likely from stroke involving the deep subcortical structures. In patients presenting with acute dystonia, particularly with associated symptoms such as weakness in the extremities, AIS should be considered in the differential diagnosis, and they should be screened for thrombolytics if they are in the treatment window.

## References

[1] Nakawah MO, Lai EC (2016). Post-stroke dyskinesias. Neuropsychiatr Dis Treat.

[2] Bansil S, Prakash N, Kaye J, Wrigley S, Manata C, Stevens-Haas C, Kurlan R (2012). Movement disorders after stroke in adults: a review. Tremor Other Hyperkinet Mov (N Y).

[3] Siniscalchi A, Gallelli L, Labate A, Malferrari G, Palleria C, Sarro GD (2012). Post-stroke movement disorders: clinical manifestations and pharmacological management. Curr Neuropharmacol.

[4] Moher D, Liberati A, Tetzlaff J, Altman DG (2009). Preferred reporting items for systematic reviews and meta-analyses: the PRISMA statement. PLoS Med.

[5] Dhakar MB, Watson C, Rajamani K (2015). Acute onset dystonia after infarction of premotor and supplementary motor cortex. J Stroke Cerebrovasc Dis.

[6] Nishimura K, Uehara T, Toyoda K (2014). Early-onset dystonia after supplementary motor area infarction. J Stroke Cerebrovasc Dis.

[7] Miletić V, Blažina K (2015). Hemidystonia caused by frontal cortical infarction. Acta Neurol Belg.

[8] Sabrie M, Berhoune N, Nighoghossian N (2015). Alien hand syndrome and paroxystic dystonia after right posterior cerebral artery territory infarction. Neurol Sci.

[9] Choi HY, Jung YJ, Shin HW (2015). Multifocal dystonia as a manifestation of acute midbrain infarction. J Neurol Sci.

[10] Kim HJ, Lee MC, Kim JS, Chung SJ, Kim HJ, Kwon M, Shin HW (2009). Lingual dystonia as a manifestation of thalamic infarction. Mov Disord.

[11] Tan EK, Chan LL, Auchus AP (2005). Hemidystonia precipitated by acute pontine infarct. J Neurol Sci.

[12] Ogawa T, Shojima Y, Kuroki T, Eguchi H, Hattori N, Miwa H (2018). Cervico-shoulder dystonia following lateral medullary infarction: a case report and review of the literature. J Med Case Rep.

[13] Zadro I, Brinar VV, Barun B, Ozretić D, Habek M (2008). Cervical dystonia due to cerebellar stroke. Mov Disord.

[14] Ghika-Schmid F, Ghika J, Regli F, Bogousslavsky J (1997). Hyperkinetic movement disorders during and after acute stroke: the Lausanne Stroke Registry. J Neurol Sci.

[15] Alarcón F, Zijlmans JC, Dueñas G, Cevallos N (2004). Post-stroke movement disorders: report of 56 patients. J Neurol Neurosurg Psychiatry.

[16] Purves D, Augustine GJ, Fitzpatrick D (2001). Circuits within the basal ganglia system. Neuroscience.

[17] Goldberg JH, Farries MA, Fee MS (2013). Basal ganglia output to the thalamus: still a paradox. Trends Neurosci.

[18] Jürgens U (1984). The efferent and afferent connections of the supplementary motor area. Brain Res.

